# Neoadjuvant radiochemotherapy is safe and feasible for breast conserving surgery or immediate reconstruction

**DOI:** 10.1038/s41598-024-59961-0

**Published:** 2024-04-22

**Authors:** Jingjing Yuan, Meilin Zhang, Maoli Wang, Mingdi Zhang, Kejin Wu, Hongliang Chen

**Affiliations:** Department of Breast Surgery, Obstetrics and Gynecology Hospital of Fudan University, Shanghai, 200011 China

**Keywords:** Chemotherapy, Radiotherapy, Breast cancer, Cancer therapy

## Abstract

This study aimed to evaluate the survival outcomes of neoadjuvant radiochemotherapy (NARCT) for early breast cancer. Female patients ≤ 80 years old with unilateral T1-T4 invasive ductal breast cancer treated with neoadjuvant chemotherapy (NAC) and radiation therapy (RT) between 2006 and 2015 were enrolled from SEER database. Baseline differences in clinical and pathological characteristics were evaluated using chi-square test. The survival outcomes were estimated by Kaplan–Meier analysis and compared using Cox hazards models. The effects of baseline differences on survival outcome in patients treated with neoadjuvant radiation therapy (NART) and post-operation radiation therapy (PORT) were circumvented by propensity score matching (PSM). Altogether 14,151 patients receiving NAC and RT were enrolled, among whom 386 underwent NART. Based on a 1:4 PSM cohort, NART was an independent unfavorable prognostic factor for breast cancer-specific survival (BCSS) and overall survival (OS) for the whole cohort. However, among patients receiving breast conserving surgery (BCS) (HR 1.029, P = 0.915 for BCSS; HR 1.003, P = 0.990 for OS) or implant-based immediate breast reconstruction (IBR) (HR 1.039, P = 0.921 for BCSS; HR 1.153, P = 0.697 for OS), those treated with NART had similar survival outcomes compared with patients treated with PORT. In conclusion, NARCT was a safe and feasible approach for patients undergoing BCS and IBR.

## Introduction

Neoadjuvant chemotherapy (NAC) has proven effective for decreasing tumor size before breast conserving surgery (BCS) and immediate breast reconstruction (IBR). Recently, there has been growing interest in treating patients with radiotherapy (RT) prior to breast surgery, that is, neoadjuvant radiotherapy (NART). As we know, postoperative radiotherapy (PORT) has been shown to decrease locoregional recurrence and improve survival outcomes in patients undergoing breast conserving surgery and those with locally advanced breast cancers (LABC)^1^.

However, NART has not been widely applied in breast cancer although it is routinely used for other radiosensitive cancers. In breast cancer, NART consists of external beam radiotherapy to the breast, supraclavicular fossa, and level III axillary nodal volumes. Further nodal coverage (internal mammary nodal and level I-II axilla) may be undertaken at the discretion of the treating radiation oncologist^2^. Historically, NART was administered in the setting of inoperable breast cancer, i.e., LABC. However, the potential advantages of NART include accurate tumor site identification, tumor down-staging to increase the feasibility of BCS, and avoiding tissue flaps and expander irradiation after breast reconstruction. NART may also reduce tumor volume preoperatively to facilitate BCS and has been shown to facilitate the indications of conservative treatment in patients with a large primary breast cancer^3^. Moreover, patients have reported high levels of satisfaction with the cosmetic outcome of BCS following NART^4^. NART aims to improve aesthetic results and simplify the reconstructive pathway without an increase in surgical morbidity and reconstruction complications^5^. Ultimately, NART affords the benefits of IBR without concerns for delayed adjuvant therapy^6^. It is worthwhile exploring the clinical significance of NART in breast cancer treatment, especially in combination with NAC, that is, neoadjuvant radiochemotherpay (NARCT). At present, there is a paucity of literature on NARCT with respect to feasibility and oncological safety.

In this study, we aimed to evaluate the survival outcomes of NARCT compared with traditional NAC followed by PORT in early-stage breast cancer patients undergoing BCS and IBR.

## Results

### Baseline characteristics of the study cohort

In total, 14,515 patients receiving NAC and RT between 2006 and 2015 were enrolled. Altogether 5881 patients (40.5%) had stage III disease, 4901 (33.8%) had T3 or T4 disease, 10,039 (69.2%) had positive lymph nodes, and 8526 (58.7%) had negative ER status. Among them, 386 (2.7%) underwent NART while 14,129 (97.3%) underwent PORT. Compared with patients undergoing PORT, those undergoing NART had a higher proportion of AJCC IIIB-IIIC, T4, and ER, PR negative cases, and were more likely to have undergone mastectomy (Table 1).

**Table 1:** Comparison of baseline characteristics between patients receiving NART and PORT in the setting of NAC in the whole cohort and matched cohort.

	Whole Cohort			Matched Cohort		
	NART	PORT	*P*	NART	PORT	*P*
Age stage			0.885			0.603
≤ 50	190 (49.2%)	6902 (48.8%)		184 (49.3%)	726 (50.8%)	
> 50	196 (50.8%)	7227 (51.2%)		189 (50.7%)	702 (49.2%)	
Race			0.159			0.291
White	275 (71.2%)	10,147 (71.8%)		266 (71.3%)	1058 (74.1%)	
Black	77 (19.9%)	2410 (17.1%)		75 (20.1%)	238 (16.7%)	
Others*	34 (8.8%)	1572 (11.1%)		32 (8.6%)	132 (9.2%)	
Histologic grade			0.875			0.602
I	13 (3.4%)	548 (3.9%)		13 (3.5%)	62 (4.3%)	
II	127 (32.9%)	4606 (32.6%)		122 (32.7%)	437 (30.6%)	
III	246 (63.7%)	8975 (63.5%)		238 (63.8%)	929 (65.1%)	
AJCC stage			< 0.001			0.796
I	32 (8.3%)	1068 (7.6%)		32 (8.6%)	91 (6.4%)	
IIA	73 (18.9%)	3468 (24.5%)		72 (19.3%)	277 (19.4%)	
IIB	93 (24.1%)	3900 (27.6%)		92 (24.7%)	371 (26.0%)	
IIIA	79 (20.5%)	2925 (20.7%)		78 (20.9%)	307(21.5%)	
IIIB	69 (17.9%)	1583 (11.2%)		63 (16.9%)	241 (16.9%)	
IIIC	40 (10.4%)	1185 (8.4%)		36 (9.7%)	141 (9.9%)	
T			< 0.001			0.224
T1	59 (15.3%)	2471 (17.5%)		59 (15.8%)	193 (13.5%)	
T2	163 (42.2%)	6921 (49.0%)		161 (43.2%)	614 (43.0%)	
T3	73 (18.9%)	2829 (20.0%)		72 (19.3%)	330 (23.1%)	
T4a–c	57 (14.8%)	1098 (7.8%)		50 (13.4%)	152 (10.6%)	
T4d	34 (8.8%)	810 (5.7%)		31 (8.3%)	139 (9.7%)	
N			0.350			0.592
N0	125 (32.4%)	4351 (30.8%)		121 (32.4%)	423 (29.6%)	
N1	165 (42.7%)	6587 (46.6%)		160 (42.9%)	665 (46.6%)	
N2	56 (14.5%)	2006 (14.2%)		56 (15.0%)	199 (13.9%)	
N3	40 (10.4%)	1185 (8.4%)		36 (9.7%)	141 (9.9%)	
Breast surgery			< 0.001			0.896
BCS	123 (31.9%)	6105 (43.2%)		123 (33.0%)	476 (33.3%)	
Mastectomy	263 (68.1%)	8024 (56.8%)		250 (67.0%)	952 (66.7%)	
Axillary surgery			0.005			0.882
No LN removed	36 (9.3%)	776 (5.5%)		25 (6.7%)	89 (6.2%)	
Biopsy	191 (49.5%)	7075 (50.1%)		190 (50.9%)	716 (50.1%)	
Dissection	159 (41.2%)	6278 (44.4%)		158 (42.4%)	623 (43.6%)	
ER			0.023			0.282
Negative	181 (46.9%)	5808 (41.1%)		173 (46.4%)	618 (43.3%)	
Positive	205 (53.1%)	8321 (58.9%)		200 (53.6%)	810 (56.7%)	
PR			0.016			0.169
Negative	229 (59.3%)	7506 (53.1%)		217 (58.2%)	774 (54.2%)	
Positive	157 (40.7%)	6623 (46.9%)		156 (41.8%)	654 (45.8%)	
HER2 (since 2010)			0.316			0.707
Negative	171 (69.2%)	6949 (66.2%)		170 (69.1%)	665 (67.9%)	
Positive	76 (30.8%)	3551 (33.8%)		76 (30.9%)	315 (32.1%)	

*American Indian/AK native, Ascian/Pacific Islander.

### Survival outcomes between patient undergoing NARCT and NAC with PORT based on PSM

**Figure 1:**
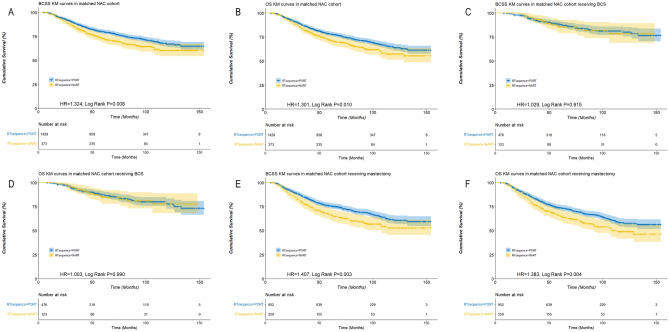
Survival curves of breast cancer-specific survival (BCSS) and overall survival (OS) stratified by neoadjuvant radiation therapy (NART) or post-operation radiation therapy (PORT) in patients receiving neoadjuvant chemotherapy based on the matched cohorts [(**A** NART was associated with a lower BCSS in the matched cohort; (**B**) NART was associated with a lower OS in the matched cohort; (**C**) NART was associated with a similar BCSS in patients undergoing BCS; (**D**) NART was associated with a similar OS in patients undergoing BCS; (**E**) NART was associated with a lower BCSS in patients undergoing mastectomy; (**F**) NART was associated with a lower OS in patients undergoing mastectomy].

Due to the significant differences in sample size and clinical-pathological characteristics between NART and PORT, a 1:4 propensity score matching (PSM) was conducted successfully for the purpose of balancing (Table 1). After the 1:4 case control matching by PSM, 373 NART cases were matched with 1428 PORT cases (Table 1). NART cases had a significantly lower BCSS (HR 1.324, Log Rank P = 0.008) and OS (HR 1.301, Log Rank P = 0.010) compared with PORT cases on a whole (Fig. 1A,B). NART was an independent unfavorable factor associated with BCSS (HR 1.272, 95% CI: 1.029–1.573, P = 0.026) and OS (HR 1.239, 95% CI: 1.010–1.519, P = 0.040) (Table 2).

**Table 2:** Multivariate Cox analysis of prognostic factors for BCSS and OS in the matched cohort.

	BCSS			OS		
	HR	95% CI	P	HR	95% CI	P
Year of diagnosis	0.976	0.911–1.046	0.489	0.960	0.899–1.026	0.230
Age						
> 50y vs. ≤ 50y	1.183	0.981–1.428	0.079	1.256	1.049–1.503	0.013
Race			0.072			0.032
Black vs. White	1.241	0.983–1.566	0.069	1.274	1.020–1.590	0.032
Others*vs. White	0.803	0.551–1.170	0.254	0.797	0.554–1.146	0.221
Histologic grade			0.006			0.027
II vs. I	1.187	0.652–2.160	0.575	0.998	0.591–1.686	0.995
III vs. I	1.672	0.927–3.015	0.088	1.331	0.794–2.229	0.278
T			0.061			0.102
T2 vs. T1	1.134	0.817–1.572	0.453	1.011	0.748–1.367	0.944
T3 vs. T1	1.277	0.900–1.811	0.171	1.148	0.831–1.585	0.402
T4a-c vs. T1	1.549	1.064–2.256	0.023	1.390	0.980–1.970	0.065
T4d vs. T1	1.568	1.060–2.320	0.024	1.373	0.952–1.981	0.090
N			< 0.001			
N1 vs. N0	1.545	1.179–2.025	0.002	1.591	1.228–2.061	< 0.001
N2 vs. N0	2.696	1.961–3.706	< 0.001	2.733	2.015–3.709	< 0.001
N3 vs. N0	3.136	2.232–4.405	< 0.001	3.101	2.235–4.304	< 0.001
Breast surgery						
Mastectomy vs. BCS	1.654	1.271–2.153	< 0.001	1.715	1.332–2.207	< 0.001
Axillary surgery			0.207			0.106
None vs. dissection	1.383	0.954–2.005	0.087	1.449	1.019–2.060	0.039
Biopsy vs. dissection	0.995	0.809–1.225	0.965	1.007	0.826–1.228	0.944
Radiotherapy						
NART vs. PORT	1.272	1.029–1.573	0.026	1.239	1.010–1.519	0.040
ER						
Positive vs. negative	0.795	0.618–1.024	0.075	0.763	0.598–0.972	0.029
PR						
Positive vs. negative	0.701	0.542–0.908	0.007	0.709	0.553–0.908	0.006
HER2						
Positive vs. negative	0.573	0.421–0.780	< 0.001	0.625	0.468–0.836	< 0.001

*American Indian/AK native, Ascian/Pacific Islander.

### Survival outcomes between patient undergoing NARCT and NAC with PORT who were treated with BCS or IBR

Based on the matched cohort, among patients undergoing BCS, those treated with NART had similar BCSS (HR 1.029, Log Rank P = 0.915) and OS (HR 1.003, Log Rank P = 0.990) compared with those treated with NAC and PORT (Fig. 1C,D). On the contrary, among patients undergoing mastectomy, those treated with NART had significantly lower BCSS (HR 1.407, Log Rank P = 0.003) and OS (HR 1.383, Log Rank P = 0.004) compared with those treated with PORT (Fig. 1E,F).

**Figure 2:**
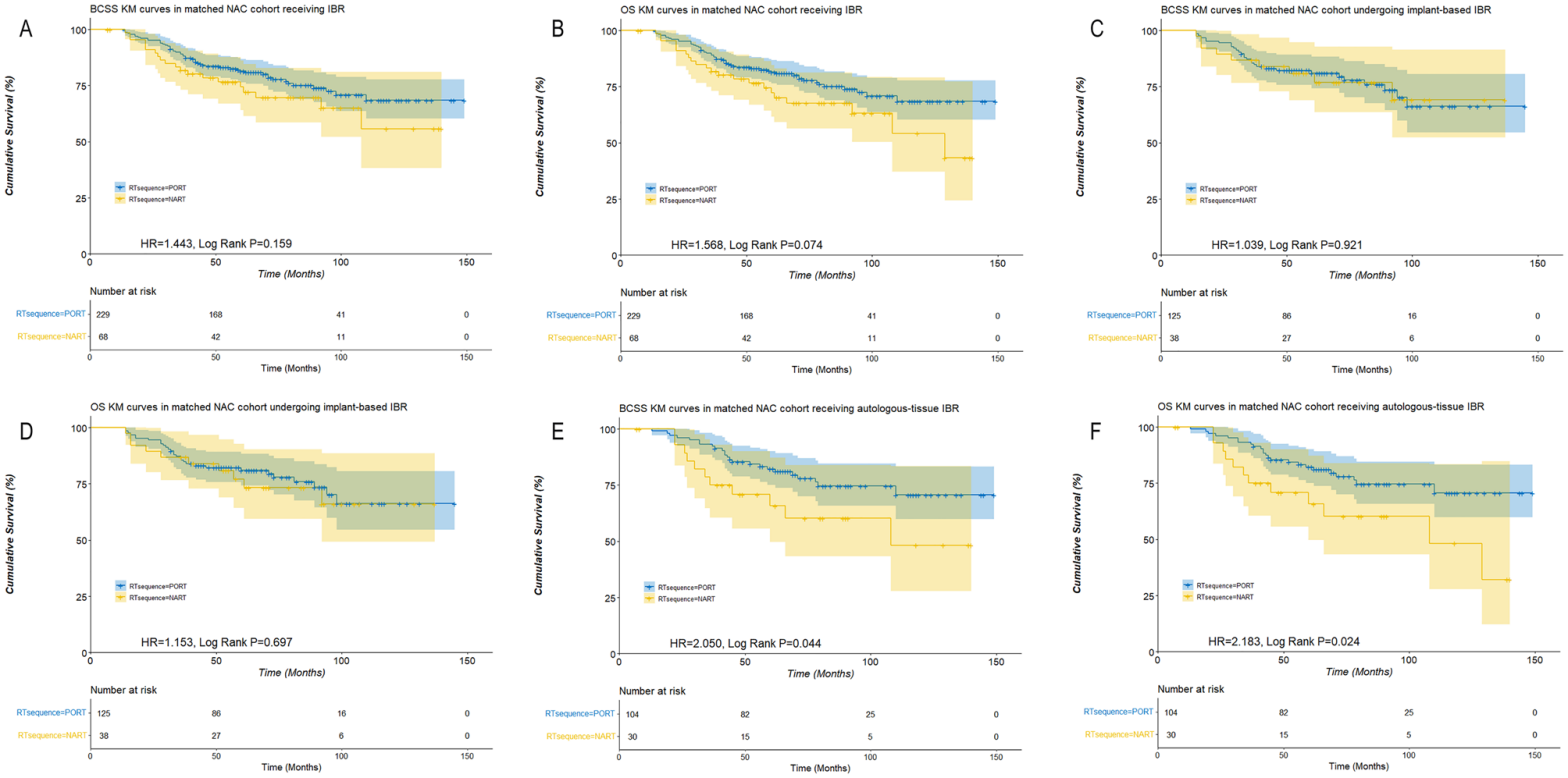
Survival curves of breast cancer-specific survival (BCSS) and overall survival (OS) stratified by neoadjuvant radiation therapy (NART) or post-operation radiation therapy (PORT) in patients receiving neoadjuvant chemotherapy undergoing immediate breast reconstruction (IBR) based on the matched cohorts [(**A)** NART was associated with a tendency towards lower BCSS in the whole matched cohort; (**B**) NART was associated with a tendency towards lower OS in the whole matched cohort; (**C**) NART was associated with a similar BCSS in patients undergoing implant-based IBR; (**D**) NART was associated with a similar OS in patients undergoing implant-based IBR; (**E**) NART was associated with a lower BCSS in patients undergoing autologous-tissue reconstruction; (**F**) NART was associated with a lower OS in patients undergoing autologous-tissue reconstruction].

Among 2475 patients receiving NAC followed by IBR, 74 cases (32 cases with autologous-tissue reconstruction and 42 cases with implant-based reconstruction) were treated with NART and 2401 were treated with PORT. After a 1:4 case control matching by PSM, 68 NART cases were matched with 229 PORT cases. NART followed by IBR demonstrated a tendency towards lower BCSS (HR 1.443, Log Rank P = 0.159) and OS (HR 1.568, Log Rank P = 0.074) compared with PORT (Fig. 2A,B). However, in the subgroup analyses, NART followed by implant-based reconstruction was associated with similar BCSS (HR 1.039, Log Rank P = 0.921) and OS (HR 1.153, Log Rank P = 0.697) as PORT (Fig. 2C,D), while for cases undergoing autologous-tissue reconstruction, those treated with NART had significantly lower BCSS (HR 2.050, Log Rank P = 0.044) and OS (HR 2.183, Log Rank P = 0.024) compared with PORT (Fig. 2E,F).

## Discussion

To our knowledge, this study is among the largest to address the significance of NARCT for breast cancer treated with BCS and IBR. We enrolled patients receiving NAC based on the following considerations. Firstly, NAC is the standard care for LABC. It is of clinical significance to evaluate NART only under the standard treatment mode. Meanwhile, the reception of NAC could exclude to the largest extent the possibility that some patients could not bear NAC and could only bear NART for local control due to significant comorbidities. NART is currently under investigation for the treatment of breast cancer^7^. The application of pre-operative radiotherapy in breast cancer may be advantageous due to more accurate identification of local tumor extension compared with post-surgical bed, a higher probability of pathological complete response (pCR) following neoadjuvant treatments^8,9^, facilitation of BCS or IBR^3,9–11^, partial breast irradiation, and to aid translational research. Our results suggest that NARCT may be feasible and safe in terms of survival outcomes for patients undergoing BCS and implant-based reconstruction.

Only one large randomized trial has so far compared NART with PORT. After a mean follow-up period of 16 years, there was no significant local recurrence between patients treated with PORT or NART in any of the analyzed endpoints^12^. Koenig et al. indicated that rates of unplanned readmissions were similar in patients treated with NART or PORT based on the National Cancer Database (NCDB)^13^. Delayed surgery after NART due to the potential development of fibrosis is a potential concern. However, a period of 4–16 weeks between NARCT and surgery did not result in a higher risk of surgical complications, and showed favorable aesthetic outcomes^2,14–16^.

Multiple studies have demonstrated favorable postoperative complication rates among BCS and IBR cases treated with NART^4–6,17–19^. Theoretically, NARCT could reduce postoperative complication rates caused by PORT and improve long-term quality of life^20^. A systemic review demonstrated that NART was oncological safe and technically feasible in the setting of IBR, with most patients rating their cosmetic outcomes as good or excellent^21^. In addition, the overall cosmetic result in patients treated with NARCT who underwent BCS was rated as "excellent" or "good" in 80% of cases^22^. Overall, our study focused on the survival outcomes of NARCT in the setting of BCS and IBR.

Survival outcomes in patients treated with NART showed contradictory results, which may be due to bias as a result of the low number of cases^8,23,24^. A recent retrospective study including 76 patients with inoperable LABC confirmed that NART was effective in downstaging inoperable LABC for surgical resection^25^. However, a NCDB study showed that NART was not associate with better survival than PORT in patients with LABC^26^. Another study showed that NART reduced the risk of second primary cancer among estrogen receptor-positive patients without decreasing overall survival^8^.

When RT was administered prior to surgery, it was applied mainly for local control and tumor down-staging. NART was previously shown to have efficacy in combination with NAC^23^. NAC for luminal-like, node positive breast cancer presented relatively low rates of pCR (16.6%), and pCR significantly increased the probability to receive BCS (42.0%)^27^. Roth et al. demonstrated that BCS became possible in 50.8% of patients following NARCT for LABC with a pCR rate of 29.2%^14^. For patients with LABC who were resistant to primary chemotherapy, 71 out of 120 patients underwent conservative treatment following NARCT^28^. Matuschek et al. evaluated 315 patients with LABC treated with NARCT with a pCR rate of 29.2% in both breast and axilla, and showed that NARCT and pCR were independent factors for better OS^16^. A pooled analysis by Adams et al. showed that NARCT achieved a pCR in 34% patients that translated into superior disease-free survival (DFS) and OS^29^. For patients with negative HR, additional chemotherapy may be added depending on whether there is residual cancer after NAC or surgery. The higher pCR in the group of patients who underwent NARCT may have required less additional adjuvant treatment compared to the PORT group. Concurrent RT may also improve the tumor response to NAC, especially in chemo-refractory tumors^30^, which may be associated with antitumor immune mechanisms^31–34^, however, more research is needed. According to a study by Deng et al., patients with LABC who received NART combined with neoadjuvant systemic treatments experienced higher survival rates when compared with those not treated with NART^26^. However, this study showed that NARCT had similar survival outcomes compared with PORT when followed by BCS, while poorer survival outcomes were observed when followed by mastectomy. There may be some potential explanation. Good response to neoadjuvant treatment significantly increased the probability to receive BCS. A higher pCR rate or obvious tumor response through neoadjuvant treatment (NARCT or NAC) will not only translate into a higher rate of BCS, but also predict improvement in prognosis. On the contrary, patients undergoing mastectomy were expected to have a lower pCR rate or a higher rate of poor tumor response. In such cases, a timely surgery followed by required adjuvant systemic therapy should be of priority, and RT could be postponed. NARCT delayed surgery and additional adjuvant systemic treatment compared with PORT, which might have a negative impact on the survival outcomes. Besides, concurrent RT may be delivered for the purpose of improving response to NAC. But if the tumor still showed poor response to concurrent NAC and NART, it was expected to have even poorer prognosis than resistance to NAC only.

Several studies reported the efficacy of NARCT as a precursor for successful IBR^18,21,35,36^, and demonstrated comparable reconstruction-associated morbidity with PORT and a trend for improved survival^5,36,37^. In this study, NARCT was oncological safe in patients undergoing implant-based reconstruction. It could also be postulated that patients who responded well to NARCT might choose implant-based reconstruction for postoperative cosmetic improvement. On the contrary, for some patients, especially those with T4 disease, NART might be performed in the case of skin nodules, ulceration, edema, or rupture when poor response to neoadjuvant systemic therapy is observed. Autologous tissue reconstruction may also be performed for the purpose of covering any defects after mastectomy. As a result, poor prognosis could be expected.

It will be important to explore potential biomarkers of response to NARCT. Research has suggested that gene expression changes are induced by radiation therapy, mainly in the p53 signaling pathway^38^. In addition, MAP3K4 may be a putative biomarker of response to PRRT^39^, which could help to optimize NART for localized treatment and may warrant further exploration.

There are some limitations to our study. Firstly, the retrospective nature of the study may have introduced bias as not all variables could be controlled fully. Information on tumor response to neoadjuvant therapy was unavailable, and the medical reasons for patients undergoing NARCT were not recorded in the SEER database. In addition, the effect of HER2 status and endocrine therapy could not be evaluated due to a lack of information. Secondly, information on locoregional recurrence was unavailable, which was an important factor of oncological safety. However, locoregional recurrence is strongly correlated with distant metastasis and survival outcomes. According to EBCTCG meta-analysis, RT after BCS reduced the 10-year risk of locoregional and distant recurrence, and reduced the 15-year risk of breast cancer death^40^. Thirdly, although PSM was adopted to reduce imbalance and bias between the two groups, it could only control the influence of measurable variables and hidden bias may have occurred with regards to other variables. Lastly, information on postoperative complications after RT was unavailable in the SEER database, which was a key limitation.

In conclusion, NARCT may be a safe and feasible alternative procedure for patients with early breast cancer, particularly for patients undergoing BCS and IBR. Large clinical trials are warranted to confirm these findings.

## Methods

### Study cohorts and stratifications

**Figure 3:**
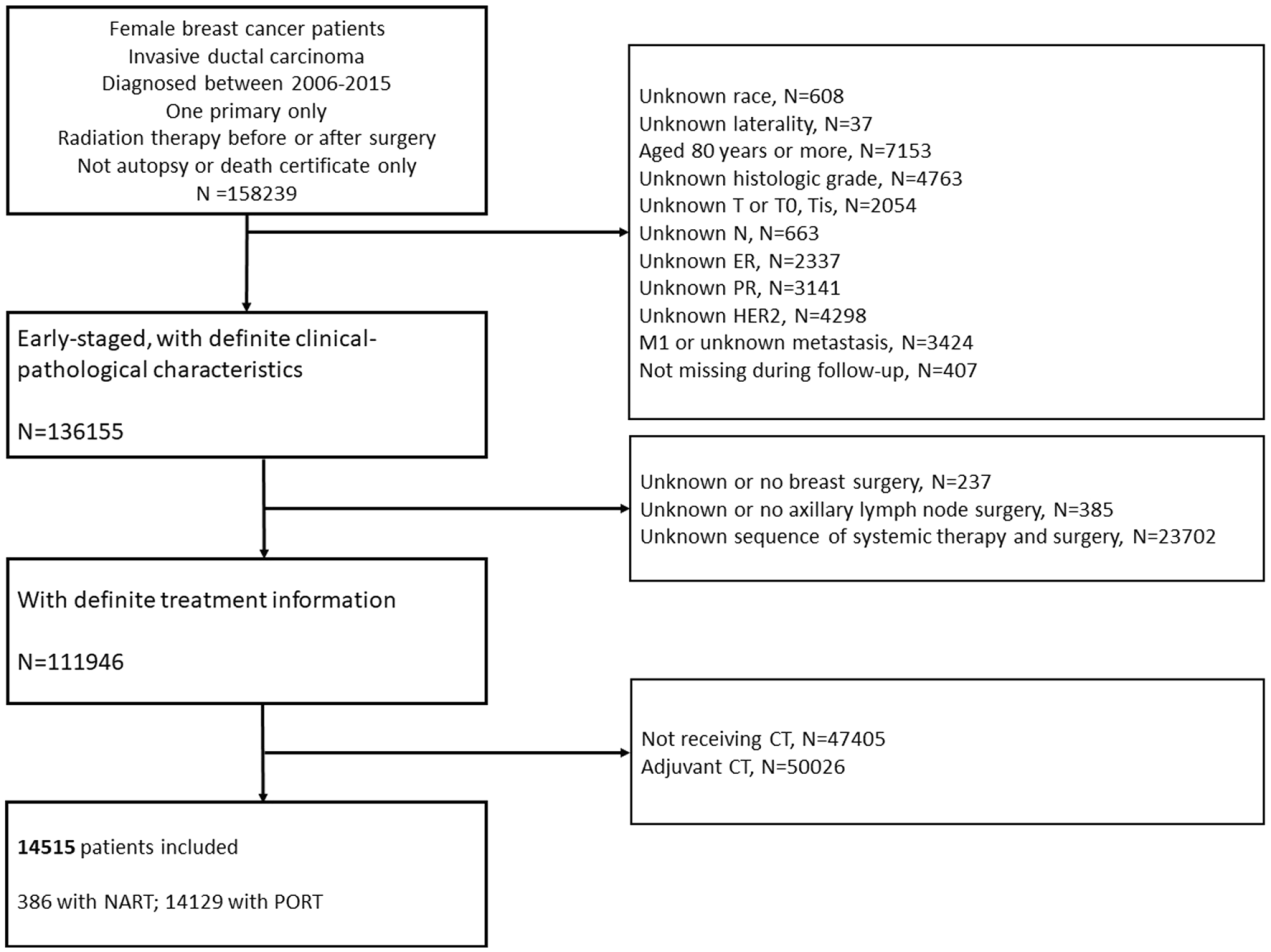
Flow chart detailing patient enrollment.

We used patient data derived from the Surveillance, Epidemiology, and End Results (SEER) database released in 2020. Female patients with unilateral primary invasive ductal carcinoma (IDC) treated with neoadjuvant chemotherapy (NAC) and radiation therapy (RT) between 2006 and 2015 were enrolled. Patients who had more than one primary cancer, metastatic disease at diagnosis, no surgery performed or no record of surgery, diagnosed at death or autopsy alone, or lost to follow up were excluded. Patients aged over 80 years, those with T0 disease, unknown race, laterality, histologic grade, T or N category, ER or PR or HER2 status, and unknown sequence of chemotherapy (CT) or RT with surgery were also excluded. The patient cohort selection flow chart is shown in Fig. 3. Cases diagnosed between 2004 and 2009 were based on AJCC 6th edition criteria and cases diagnosed between 2010 and 2015 were based on AJCC 7th edition criteria. Histologic grade III was defined as poorly differentiated and anaplastic histologic grade disease. ER or PR borderline status was considered as unknown. CT prior to surgery was defined as NAC. RT prior to surgery was defined as NART while PORT was known as adjuvant RT. Patients undergoing NAC and NART were defined as NARCT. The SEER database is an open public database that does not include personal information; therefore, informed consent was not required.

### Statistical analysis

Clinical-pathological characteristics in patient who underwent NART and PORT were compared by Pearson’s Chi square test. Follow-up data up to 31 December 2018 were included. Breast cancer-specific survival (BCSS) was defined as the interval from breast cancer diagnosis to death from breast cancer or the last follow-up. Overall survival (OS) was defined as the interval from diagnosis to death from any cause or the last follow-up. BCSS and OS were compared across NART and PORT by means of Kaplan–Meier analysis and log-rank tests. Significant independent prognostic factors were evaluated by a Cox hazards model and presented as adjusted hazard ratios (HRs) with 95% confidence intervals (CIs). In survival outcomes comparison, the propensity score matching (PSM) method was adopted. PSM attempts to approximate a completely randomized experiment. It can reduce imbalance and bias between the treated and control groups, allowing for a more reasonable comparison, especially when there is a significant difference in sample size between the two groups. In this study, a 1:4 PSM matching was conducted. Factors including year stage at diagnosis, age, race, histologic grade, T and N stage, breast surgery (breast conserving surgery and mastectomy, implant, or autologous tissue reconstruction), axillary surgery, and ER, PR, HER2 status were well balanced, among which breast surgery and AJCC stage were exactly matched (Table 1). All statistical tests were two sided, and statistical significance was defined as P < 0.05. SPSS 22.0 and R-statistics 4.12 were used for statistical calculations.

### Ethics declarations and consent to participate

All procedures performed in studies involving human participants were in accordance with the ethical standards of the institutional and/or national research committee and with the 1964 Helsinki declaration and its later amendments or comparable ethical standards. This article does not contain any studies with animals performed by any of the authors. As SEER database is an open public database without involving personal information, informed consent was consequently not required. The Obstetrics and Gynecology Hospital of Fudan University IRB has reviewed the project and has determined this project does not meet the definition of human subject research under the purview of the IRB according to the national regulations.

## Data Availability

Publicly available datasets were analyzed in this study. The data can be found here: https://seer.cancer.gov/data/.
